# Quantifying breast milk intake by term and preterm infants for input into paediatric physiologically based pharmacokinetic models

**DOI:** 10.1111/mcn.12938

**Published:** 2020-01-21

**Authors:** Cindy H.T. Yeung, Simon Fong, Paul R.V. Malik, Andrea N. Edginton

**Affiliations:** ^1^ School of Pharmacy University of Waterloo Kitchener Ontario Canada

**Keywords:** breast milk, breastfeeding, feeding frequency, pharmacokinetics, preterm infants, risk assessment, volume of intake

## Abstract

Despite the many benefits of breast milk, mothers taking medication are often uncertain about the risks of drug exposure to their infants and decide not to breastfeed. Physiologically based pharmacokinetic models can contribute to drug‐in‐milk safety assessments by predicting the infant exposure and subsequently, risk for toxic effects that would result from continuous breastfeeding. This review aimed to quantify breast milk intake feeding parameters in term and preterm infants using literature data for input into paediatric physiologically based pharmacokinetic models designed for drug‐in‐milk risk assessment. Ovid MEDLINE and Embase were searched up to July 2, 2019. Key study reference lists and grey literature were reviewed. Title, abstract and full text were screened in nonduplicate. Daily weight‐normalized human milk intake (WHMI) and feeding frequency by age were extracted. The review process retrieved 52 studies. A nonlinear regression equation was constructed to describe the WHMI of exclusively breastfed term infants from birth to 1 year of age. In all cases, preterm infants fed with similar feeding parameters to term infants on a weight‐normalized basis. Maximum WHMI was 152.6 ml/kg/day at 19.7 days, and weighted mean feeding frequency was 7.7 feeds/day. Existing methods for approximating breast milk intake were refined by using a comprehensive set of literature data to describe WHMI and feeding frequency. Milk feeding parameters were quantified for preterm infants, a vulnerable population at risk for high drug exposure and toxic effects. A high‐risk period of exposure at 2–4 weeks of age was identified and can inform future drug‐in‐milk risk assessments.

**Key messages**

- Physiologically based pharmacokinetic (PBPK) models can be used to predict infant exposure as part of drug‐in‐milk risk assessments.
- Existing reviews approximating breast milk intake for input into these PBPK models are unable to address exclusive breastfeeding across all ages, preterm infants and feeding frequency.
- Results from this literature review were used to construct a nonlinear regression equation on the weight‐normalized human milk intake and determine a weighted mean feeding frequency of exclusively breastfed term infants.
- Preterm infants fed with similar feeding parameters to term infants on a weight‐normalized basis.
- A high‐risk period of exposure at 2–4 weeks of age was identified.

## 1 INTRODUCTION

Breastfeeding is the accepted standard for infant feeding and nutritional support and has been linked to improved health outcomes and neurodevelopmental advantages in both developed and developing countries. The American Academy of Pediatrics (AAP) and the World Health Organization (WHO) recommend that infants be exclusively breastfed for the first 6 months postpartum, after which complementary foods can slowly be introduced (Eidelman & Schanler, 2012). Advantages of breastfeeding include reduced incidence and severity of respiratory tract infections and otitis media in the newborn (Chantry, Howard, & Auinger, 2006; Ip et al., 2007; Ip, Chung, Raman, Trikalinos, & Lau, 2009), and protection against allergic disease states (Greer, Sicherer, & Burks, 2008; Ip et al., 2007) and metabolic disorders such as obesity and diabetes later in life (Das, 2007; Mayer et al., 1988; Owen, Martin, Whincup, Smith, & Cook, 2005; Rosenbauer, Herzig, & Giani, 2008). Neurodevelopmental outcomes of preterm neonates who were breastfed have also shown improvement compared with their counterparts, as demonstrated by greater white matter and total brain volume, and increased intelligence quotients (Isaacs et al., 2010).

Despite the many apparent benefits of breast milk, mothers taking medication often have difficulty deciding whether to breastfeed their infant. Uncertainty regarding the safety of breastfeeding while on medication has been frequently cited as a reason for mothers not to initiate or continue breastfeeding (Li, Fein, Chen, & Grummer‐Strawn, 2008; Nordeng, Koren, & Einarson, 2010; Teich, Barnett, & Bonuck, 2014). Concerned mothers may choose not to breastfeed due to the risk of exposing the infant to the drug through milk, which has led to serious toxicity, including death in some reported cases (Beauchamp, Hendrickson, Horowitz, & Spyker, 2019; Koren, Cairns, Chitayat, Gaedigk, & Leeder, 2006; Madadi et al., 2009; Schultz, Kostic, & Kharasch, 2019). Alternatively, mothers may discontinue taking their medication even though the resultant infant exposure to medications may actually be low. As examples, breastfeeding mothers have been shown to be noncompliant to oral antibiotics (Ito, Koren, & Einarson, 1993) and to antidepressant therapies that may have been relatively safe for the infant after a risk‐benefit ratio assessment (Boath, Bradley, & Henshaw, 2004).

As a strategy to reduce uncertainties surrounding maternal drug use during breastfeeding, risk assessments can be performed. In these assessments, an infant dose is estimated to determine the amount of drug an infant would ingest through milk (Hale & Rowe, 2016). Integrating the infant dose with a physiologically based pharmacokinetic (PBPK) model can lead to a metric of exposure that, when linked to a measure of safety, forms the basis for the risk assessment.

The incorporation of PBPK models in the process of drug development has become increasingly prevalent in the past two decades (Rowland, Peck, & Tucker, 2011). PBPK models have the ability to provide in silico estimates of drug exposure given the proper parameterization with host physiology and drug properties (Maharaj & Edginton, 2014). In order to fully exploit the utility of PBPK models in quantifying drug uptake in breastfed neonates, an accurate measure of infant feeding parameters, volume and frequency of maternal milk intake, is needed. Essentially, dose to the infant through breast milk is calculated by multiplying daily volume of milk intake by the drug concentration in milk (Anderson & Sauberan, 2016). Knowledge of the total daily milk intake combined with information on intake frequency can help identify the peak serum concentration an infant would receive after feeding (*C*_max_) and contribute to an assessment of drug safety. Total daily milk intake divided by the frequency gives volume per feed, which essentially determines the dose of the xenobiotic to the infant. Higher doses lead to higher peak concentrations and may factor in to a decision about risk during breastfeeding. The daily milk intake volume of 150 ml/kg is commonly used to determine infant dose, a value first proposed by Wilson, Brown, Hinson, and Dailey (1985) in 1983 and solidified by the WHO in 1988 (Bennett, 1988). However, as suggested by Anderson and Sauberan (2016), and clearly demonstrated in longitudinal data from the United States (Neville et al., 1988), feeding volumes are not constant across postnatal ages (PNAs) and often have large interindividual variability. It is therefore fundamental to capture representative intake volumes to inform more appropriate risk assessments.

Although feeding volumes have been captured by several reviews and reports (Butte, Lopez‐Alarcon, Garza, & Expert Consultation on the Optimal Duration of Exclusive Breastfeeding, 2002; da Costa et al., 2010; Galpin et al., 2007; Hester, Hustead, Mackey, Singhal, & Marriage, 2012; Reilly, Ashworth, & Wells, 2005; U.S. EPA, 2011), they focus solely on term infants, whereas milk intake by preterm neonates remains an unexplored area. Notwithstanding the lack of reviews for preterm infants, the need to study this population in relation to breast milk and maternal medication should be emphasized. Breast milk is particularly beneficial to preterm infants in providing appropriate nutrition during their time growing ex‐utero in a crucial period of accumulating nutrient reserves typical for the developing fetus (Bouyssi‐Kobar et al., 2016; Carlson & Ziegler, 1998; Ehrenkranz et al., 2006) and reducing necrotizing enterocolitis and sepsis, which is more prevalent in this population (Underwood, 2013). Further attention to preterm infants is also warranted because they are more vulnerable than term infants to toxicity from drug exposure through breast milk. Preterm infants have reduced capacities for drug metabolism in the liver and drug excretion in the kidneys, and as a result, eliminate drugs more slowly from the body (Kearns et al., 2003; Reiter, 2002). In comparison to term infants, their further lowered ability to eliminate drugs may lead to high sustained drug concentrations in plasma, especially over multiple doses or feeds.

In this review, a comprehensive search of the literature was performed to retrieve estimates of human milk intake and frequency for term and preterm infants as a function of PNA, as inputs for PBPK models with the purpose of subsequent drug‐in‐milk risk assessments.

## 2 METHODS

### 2.1 Eligibility criteria

Studies reporting term or preterm infants receiving human milk with data on their volume or frequency of intake were of interest. Term infants were defined as >37 gestational age (GA) at birth, and those of ≤37 GA at birth were identified as preterm infants. For term infants, articles were included if data were provided on infants of any age who were exclusively breastfeeding (EBF) or infants >6 months PNA who were partially breastfeeding (PBF). These criteria were selected to reflect the AAP and WHO recommendations and produce conservative estimates for subsequent risk assessments. For preterm infants, articles were included if data were provided on infants who were exclusively human milk‐fed or were PBF with a diet that consisted mainly of breast milk, where information regarding the proportion of human milk and other sources of nutrients in the diet were provided. Only articles with volume data presented in relation to individual infant's body weights, presented as weight‐normalized human milk intake (WHMI), were included to reduce interinfant and intrainfant variability of daily milk consumption observed in absolute milk intake measurements. Included studies were those that measured volume and frequency of intake for at least 24 hr, as intake of breast milk tends to differ throughout the day (Kent et al., 2006). Articles were excluded if infants had significant birth complications or were otherwise unhealthy, and studies in which the intake of breast milk was influenced by the study investigators, including non‐ad libitum feedings and interventions that significantly increased the milk expression of mothers.

### 2.2 Search strategy

The search strategies consisted of MeSH headings and text words related to premature and term infants, breastfeeding and volume and frequency of intake. The Ovid MEDLINE and Embase databases were searched up to July 2, 2019. Results were limited to the English language. Complete search strategies are provided in Appendix A. In addition to the searches, reference lists of key studies on volume or frequency of breastfeeding and the grey literature were used to identify studies.

### 2.3 Study selection and data extraction

Two investigators (C. H. T. Y. and S. F.) screened title, abstract and full text for inclusion. The results were not screened in duplicate. Data extraction was performed by one investigator (C. H. T. Y.).

### 2.4 Data analysis

Daily WHMI and human milk feeding frequency from each study were presented as mean ± standard deviation (*SD*) ml/kg/day and number of feeds/day, respectively. When only a median value was reported for a feeding parameter, the mean was assumed equal to the median based on an assumption of normally distributed data as demonstrated previously with individual subject measurements in five of the included studies (Arcus‐Arth, Krowech, & Zeise, 2005). Studies reporting volume of milk intake in grams were converted to millilitres using the density of milk (1.03 g/ml). PNA in days were approximated from alternative sources of infant age, such as corrected age (CA) and GA, where appropriate. Data without quantitative summaries in the literature were digitized using Plot Digitizer (v2.6.8 by Joseph Huwaldt). Data were graphically represented and analysed using MATLAB R2018b. A sample‐size‐weighted nonlinear regression was performed to quantify the WHMI by EBF infants across all studies using the Intiquan Toolbox (IQMTools v1.2.2.2 by Henning Schmidt and colleagues). The following function containing an integrated form of a first‐order increase followed by a first order decline was selected to represent the general shape of the data using the least number of parameters:

$$\text{WHMI}=\theta_{1}\cdot \frac{\theta_{2}}{\theta_{2}-\theta_{3}}\cdot \left(e^{-\theta_{3}\cdot t}-e^{-\theta_{2}\cdot t}\right),$$

where WHMI is in ml/kg/day and *t* is age of the infant in days. The unknown parameters (*θ*_1_, *θ*_2_ and *θ*_3_) were fitted using the observed data for breast milk volume, weighted by the sample size in each dataset. The cost function to be minimized was the sum of squared error. A simulated annealing temperature‐based approach was used to explore the parameter spaces. The optimization was repeated 50 times with randomized parameter start values to confirm that a global minimum had been achieved and to explore any potential correlations between parameters.

## 3 RESULTS

**Figure 1:**
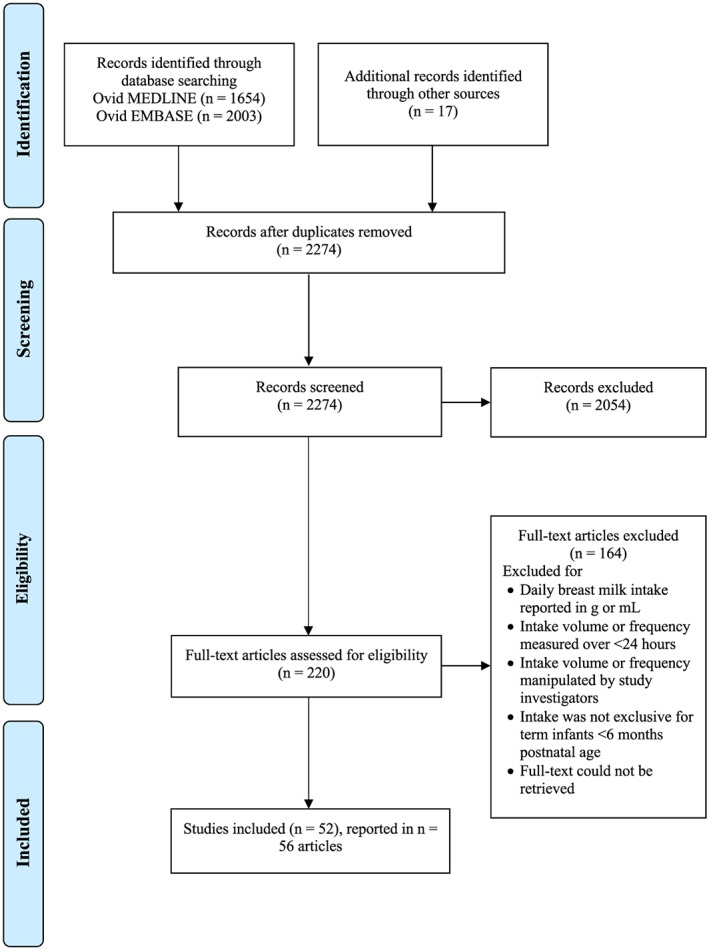
Preferred Reporting Items for Systematic Reviews and Meta‐Analyses (PRISMA) flow diagram of breast milk feeding parameter study selection

The literature search identified 2,257 nonduplicate results, and 17 articles were identified through other sources. Title and abstract screening of 2,274 records resulted in 2,054 articles excluded at this stage. In assessing the eligibility of full‐text articles, 220 results were screened and 164 were excluded. The review process resulted in 52 studies presented in 56 articles. A Preferred Reporting Items for Systematic Reviews and Meta‐Analyses (PRISMA) study selection flow diagram is provided in Figure 1, and characteristics of the included studies are described in Table 1.

**Table 1:** Characteristics of studies quantifying breast milk feeding parameters in term and preterm infants

Study	Design	Country	Main study aim	Population	Feeding parameter(s)
Aimone et al. (2009)	Longitudinal, randomized controlled	Canada	To examine the impact of feeding human milk containing extra nutrients for 12 weeks after discharge on premature infant bone mineralization, body composition and human milk use up to 1 year.	Thirty‐four preterm infants (born <33 weeks GA, 750–1,800 g; control group: 47% male, intervention group: 74% male) who completed the 12‐week randomized controlled trial by O'Connor et al. (2008) followed until 12 months CA regarding human milk feeding duration, exclusivity and time to introduction of solids.	PBF volume
Amatayakul et al. (1999)	Longitudinal, observational	Thailand	To observe whether or not previous successful breastfeeding has any influence on subsequent breastfeeding behaviour.	Sixty breastfeeding infants (42% male) who were randomly selected from three subdistricts of the rural northern Thai population, received supplementary feeding with premasticated rice within the first month of life. The infant pairs were stratified by whether their mothers were primiparous or multiparous, and were followed until 360 days of age.	PBF volume, PBF frequency
Atkinson et al. (1981)	Longitudinal, non‐randomized controlled	Canada	To examine the adequacy of preterm infant's mother's milk as compared with donor milk or formula for the premature infant.	Of the 24 LBW premature infants (born <1,300 g) selected from all patients admitted to the NICU, eight received pooled breast milk and eight received their mother's own breast milk. The infants were followed for 2 weeks during the NICU stay.	EBF volume, PBF volume
Bandara, Hettiarachchi, Liyanage, Amarasena, and Wong (2015)	Cross‐sectional, observational	Sri Lanka	To measure the human milk intake of infants during the first 6 months of age to assess their adequacy of human milk intake and to document the breastfeeding practices of their mothers.	Forty‐eight exclusively breastfeeding healthy term infants randomly selected from maternal and child health clinics. Infants were stratified across three examined age groups: <2 months, 2 to <4 months and 4–6 months.	EBF volume
Bhutta, Abbass, Wright, and Coward (2004)	Longitudinal, observational	Pakistan	To survey and evaluate the growth pattern, and breast milk and fluid intake patterns of infants in Pakistan.	Twelve term infants who were a subset of the 112 surveyed mother–infant pairs recruited from a hospital medical centre, followed over 6 months.	EBF volume
Borschel, Kirksey, and Hannemann (1986)	Longitudinal, observational	U.S.	To validate the accuracy of the test weighing procedure on volume of formula intakes for formula‐fed infants by comparing the test weighed measured values with those obtained by direct measurement.	Of the 35 healthy term infants of mothers from a university community who were followed for the first 6 months, 15 infants (67% male) were exclusively breastfed and test weighed. The infants were originally part of a larger study investigating vitamin B6 nutrition from consumed breast or formula milk.	EBF volume, EBF frequency
Brown, Robertson, and Akhtar (1986)	Longitudinal, observational	Bangladesh	To describe the amounts of breast milk‐derived macronutrients consumed by marginally nourished Bangladeshi infants and examine their patterns of growth.	Fifty‐eight infants of mothers from an underprivileged semiurban community were followed for 9 months. The infants generally had a lower body weight (approximating the U.S. National left for health Statistics (NCHS) 5th centile during the first 4 months and declining thereafter) than most exclusively breast‐fed North American infants.	EBF volume
Butte, Garza, Smith, and Nichols (1983)	Cross‐sectional, observational	U.S.	To compare the deuterium dilution technique against the test‐weighing method for measuring breast milk intake.	Twenty‐one infants who were breast‐fed exclusively (one infant customarily received water) and assessed at a mean of 3.3 ± 0.4 months of age.	EBF volume
Butte, Garza, Smith, and Nichols (1984); Butte, Garza, Stuff, et al. (1984)	Longitudinal, observational	U.S.	Butte, Garza, Smith, and Nichols (1984): To longitudinally document intakes and growth of breastfed infants to obtain normative data on human milk production and examine potential discrepancies between observed levels of human milk intake and U.S. National Center for Health Statistics (NCHS) nutrient recommendations. Butte, Garza, Stuff, et al. (1984): To examine the influence of maternal diet and body composition on lactational performance in a group of privileged, presumably well‐nourished women to establish energy recommendations during lactation.	Forty‐five exclusively breastfed healthy term infants (born 37–42 weeks GA, 2,560–4,570 g; 60% male) of mothers recruited through a milk bank program, who were observed over the first 4 months.	EBF volume, EBF frequency
Butte, Wong, Patterson, Garza, and Klein (1988)	Cross‐sectional, observational	U.S.	To compare the deuterium dilution technique against the test‐weighing method for measuring breast milk intake.	Nine term infants, with five of the infants feeding exclusively on human milk and measured at approximately 1.5, 3 and 4 months of age. The remaining four infants were fed human milk and supplemental foods.	EBF volume
Butte et al. (1990)	Cross‐sectional, observational	U.S.	To combine the doubly labelled water method with conventional indirect calorimetry to explore possible differences in energy utilization between breastfed and formula‐fed infants.	Forty term infants, of which 20 were exclusively breastfed human milk since birth. Ten of the breastfed infants were observed at 1 month, and a second set of 10 infants were observed at 4 months of age.	EBF frequency
Butte, Smith, and Garza (1990)	Cross‐sectional, observational	U.S.	To investigate the energy utilization of breastfed and formula‐fed infants, and determine whether energy utilization was different between the two feeding groups.	Sixty‐five healthy term infants whose mothers were recruited from a milk bank program were observed at 1 and 4 months of age. Of the participating infants, 32 were exclusively breastfeeding (44% male).	EBF volume
Cabrera Lafuente et al. (2018)	Longitudinal, observational	Spain	To describe the intake of mothers' own milk and its composition according to gestational age and postnatal age in preterm infants and to correlate them with neonatal weight, length and morbidities.	One‐hundred‐and‐seventy‐six preterm infants (born <32 weeks GA; 52% male) were included, where the majority of infants received partial feeding of mothers' own milk. The infants were followed at 72 hr, 15 and 30 days and monthly until discharge (90 days).	PBF volume
Carnielli et al. (1996)	Longitudinal, observational	Italy	To determine whether feeding very low birth weight infants exclusively with preterm human milk can ensure constant plasma and red blood cell long chain polyunsaturated fatty acids levels during the first month of life.	Twenty‐two preterm infants (born 750–1,750 g), of which 14 infants received mother's own milk and eight received preterm human milk, were followed for 4 weeks.	EBF volume
Casey, Neifert, Seacat, and Neville (1986)	Longitudinal, observational	U.S.	To report the intakes of milk, energy and some selected nutrients to estimate nutrient intake in the young neonate.	Eleven healthy term infants from mothers with uncomplicated pregnancies, who were put to the breast within two hours of birth and were followed for 5 days. The infants were exclusively breastfed, except for two who were given water or glucose water 3–6 times after breast feeds.	EBF volume
Cohen et al. (1994)	Longitudinal, randomized controlled	Honduras	To examine breast milk intake, total energy intake and infant growth among breastfed infants randomly assigned to receive nutritionally adequate, hygienically prepared complementary foods beginning at 16 weeks or to be exclusively breastfed until 26 weeks.	Fifty healthy term infants whose mothers were recruited from two public hospitals in Honduras. Infants were exclusively breastfed and at 16 weeks were randomly assigned to continue exclusively breastfeeding (control group), to introduce solid foods with ad libitum breastfeeding, or to introduce solid foods with maintenance of preintervention breastfeeding frequency. The infants were followed until 26 weeks of age.	EBF frequency
de Carvalho, Robertson, Merkatz, and Klaus (1982)	Longitudinal, observational	U.S.	To provide normative data for true demand breastfeeding mothers during the first 14 days postpartum, and to determine whether the frequency and duration of breastfeeding affect milk production at 1 month.	Forty‐six term infants (59% male) whose mothers were encouraged to nurse on true demand, were observed during the first 14 days after delivery.	EBF frequency
Dewey, Heinig, Nommsen, and Lonnerdal (1991a, 1991b)	Longitudinal, observational	U.S.	To present data on indexes of functional outcomes to judge whether a particular pattern of intake or growth, particularly for breastfeeding infants, is advantageous in a given environment.	Ninety‐two healthy term infants and their mothers as part of the Davis Area Research on Lactation, Infant Nutrition and Growth (DARLING) study who were followed until 12 months of age.	EBF volume, PBF volume
English (1985)	Longitudinal, observational	Australia	To report the milk production of a mother post‐partum, while considering the observed production against weight changes and activity of the mother, and breast milk intake of the infant.	One fully breastfed term infant followed for 13 weeks since birth.	EBF volume
Ettyang, van Marken Lichtenbelt, Esamai, Saris, and Westerterp (2005)	Cross‐sectional, observational	Kenya	To evaluate the association between maternal body composition and intake of breast milk in infants in Kenya.	Ten exclusively breastfed infants aged 2–4 months, from a pastoral community living in West Pokot, Kenya. The infants were a randomly selected subset of a larger longitudinal study to determine the prevalence of undernutrition, low iron stores and vitamin A deficiency during the third trimester of pregnancy and at 4 months after birth.	EBF volume
Evans, Evans, Royal, Esterman, and James (2003)	Longitudinal, observational	Australia	To determine the effect of caesarean section on breast milk transfer to the normal term infant over the first week of life.	Of the 185 mother–infant pairs, 88 term infants (50% male) from mothers who had a normal vaginal delivery were exclusively breastfed.	EBF volume
Ferris et al. (1993); Neubauer et al. (1993)	Longutidinal, observational	U.S.	Ferris et al., 1993: To examine whether differences in prenatal care and maternal health, perinatal management of lactation and infant birth outcome among women with IDDM compared with control and reference women explain the duration of human lactation and breastfeeding pattern in women with IDDM. Neubauer et al., 1993: To report the composition of breast milk from mothers with IDDM and their intake from their infants longitudinally.	Thirty‐three infants with mothers with IDDM, 33 control infants from mothers without IDDM and matched by demographic characteristics to the IDDM group, and 11 healthy reference infants followed until 84 days postpartum. Of the 11 term infants with volume of intake data, 10 infants at 7 days and nine infants at 14 days, were exclusively breastfeeding.	EBF volume, EBF frequency
Forsum and Sadurskis (1986)	Longitudinal, observational	Sweden	To examine the interactions between growth and body composition of infants, and the amount of breast milk these infants consume during the first 8–10 weeks of life.	Twenty‐two healthy breastfed infants (59% male) during the first 10 weeks of life.	EBF volume
Hendrickse et al. (1984)	Longitudinal, observational	U.K.	To describe the weight gain and calorie intake of LBW infants who were either fed predominantly on fresh raw breast milk or commercially available LBW formula.	Of the 24 preterm infants (born <33 weeks GA, <1500 g), 10 were fed predominately their own mother's fresh unpasteurized expressed breast milk and followed for their first 6 weeks of life at the NICU.	EBF volume
Hofvander, Hagman, Hillervik, and Sjolin (1982)	Cross‐sectional, observational	Sweden	To compare the amount of breast milk and breast milk substitutes consumed by 1–3 month‐old infants fed ad libitum, and living under similar conditions in their homes.	Of the 150 singleborn, healthy, term infants 75 were exclusively breastfed infants (25 infants studied per age group: at approximately 1, 2 and 3 months of age) were recruited following discharge from randomly sampling mothers from records at a maternity ward.	EBF volume
Hornell, Aarts, Kylberg, Hofvander, and Gebre‐Medhin (1999)	Longitudinal, observational	Sweden	To elucidate the variations in three components of breastfeeding pattern (frequency of feeds, suckling duration and longest interval between two consecutive feeds) in exclusively breastfed infants, while analyzing factors influencing the duration of exclusive breastfeeding and total duration of breastfeeding.	Five‐hundred‐and‐six infants were followed up from the first week after delivery until their mother's second menstruation postpartum or until a new pregnancy. At 26 weeks of follow up, seven of the original 506 infants remained exclusively breastfed. The study was part of a larger, multicentre study of duration of lactational amenorrhea in relation to breastfeeding practices in seven countries.	EBF frequency
Houston, Howie, and McNeilly (1983)	Longitudinal, observational	U.K.	To examine whether minimal intrusion by one, or at the most two, test‐weighs using an electronic balance in 24 hr would give assessments of 24‐hr feed volumes for sufficient accuracy.	Eighteen term infants (born >38 weeks GA, >2,500 g) of breastfeeding mothers were followed for the first 6 postpartum days of life.	EBF frequency
Howie et al. (1981)	Longitudinal, observational	U.K.	To report on the effect that the introduction of supplementary food may have on the frequency and duration of suckling and the resumption of ovarian activity after childbirth.	Twenty‐seven term infants who were exclusively breastfed, and had supplementary food first introduced between 3 and 24 weeks postpartum, were followed up until 40 weeks after delivery.	EBF frequency, PBF frequency
Itabashi et al. (1999)	Longitudinal, observational	Japan	To investigate the nutritional intake of ELBW infants to propose an advisable and practically feasible nutritional intake from Japanese infants with smaller average gains in body weight, head circumference and length than infants from Western countries.	Sixteen ELBW preterm infants (born 26–33 weeks GA) who were admitted to the NICU and were mainly fed preterm milk during the first 4 weeks, gradually reducing to 60% of total milk intake by the 13th week, were followed until 12 weeks of postnatal age.	PBF volume
Janas, Picciano, and Hatch (1985)	Longitudinal, observational	U.S.	To determine the relationships among specific parameters of protein, nitrogen and amino acids of term infants fed either human milk, a whey‐predominant formula, or a cow's milk formula.	Thirty‐seven term infants whose mothers were recruited at prenatal classes or immediately following their infants' birth in the hospital, were followed until 16 weeks of age. Eleven of these infants (27% male) received human milk and were introduced supplementary food after 8 weeks of age.	EBF volume
Jia et al. (2018)	Longitudinal, observational	China	To conduct a longitudinal study on tracing the growth of exclusively breastfed infants and combining human milk analysis to support breastfeeding and to achieve optimal development of infants.	Of the 130 term infants who were enrolled from seven cities in China, 59 infants (51% male) were exclusively breastfed and followed up until 6 months of age.	EBF frequency
Kent et al. (2013)	Longitudinal, observational	Australia	To assess the reproducibility of measures of breastfeeding behaviour and breast milk intake, and to provide clinicians with evidence‐based information to inform parents' expectations of their infants' breastfeeding behaviour and breast milk intake from 1 to 6 months of exclusive breastfeeding.	Fifty‐two healthy term infants who were exclusively breastfed on demand, where mother–infant pairs participated in one of four larger longitudinal studies on energy balance of lactating women, the effect of a progesterone‐only contraceptive pill on lactation, prolactin concentrations in milk and blood and the rate of milk synthesis, and breast volume and milk production during exclusive breastfeeding.	EBF frequency
Krebs, Reidinger, Robertson, and Hambidge (1994)	Longitudinal, observational	U.S.	To determine the growth pattern of normal infants who were fed human milk exclusively for at least 5 months and investigate the relationship of growth to milk intake and whether the growth was related to zinc intake from human milk.	Seventy‐one healthy term infants who were exclusively human milk fed for at least 4.5 months and were randomly assigned to receive 15‐mg zinc supplementation or placebo at 2 weeks of age (milk output between the groups were not significantly different). Of the infants, 43 continued in the study through 9 months of age.	EBF volume, PBF volume
Martinez and Chavez (1971)	Longitudinal, observational	Mexico	To obtain longitudinal data on breast milk yields of mothers from a poor rural community of the Mexican plateau under conditions of strict control and standardization.	Seventeen full term infants who were not breastfed in the first 48–72 hr of life, and were instead fed sugarless leaf infusions or donor milk. Infants were allowed to be fed on demand, including water and other supplementary foods. The mother–infant pairs were part of a larger project to understand the relationship between infant nutrition and its physical, mental and social development.	PBF volume, PBF frequency
Matheny and Picciano (1986)	Longitudinal and cross‐sectional, observational	U.S.	To determine the existence, extent and nature of relationships among nursing frequency, quantity of milk consumed and growth characteristics of exclusively breastfed infants.	Fifty healthy term infants with anthropometric measurements, milk intake and nursing frequency data acquired from three different conducted studies. Milk intake and nursing frequency data were obtained for up to 16 weeks of age.	EBF frequency
Michaelsen et al. (1994)	Prospective, observational	Denmark	To describe the nutritional role of breastfeeding and provide a detailed description of the intake, protein, fat, carbohydrate, and energy of human milk, and potential influencing factors.	Ninety‐one infants (46% male) were followed until 12 months of age and were grouped as EBF or PBF. The infants were part of a larger study, The Copenhagen Cohort study on Infant Nutrition and Growth.	EBF volume, EBF frequency, PBF volume, PBF frequency
Motil, Sheng, Montandon, and Wong (1997)	Longitudinal, observational	U.S.	To investigate the differences in nitrogen and energy utilization between breast‐ and formula‐fed infants by measuring longitudinally the differences in body composition and protein and energy intakes who were fed either human milk or a commercial formula.	Twenty term infants, of which ten were breastfed, were followed until 24 weeks postnatal age.	EBF volume
Neville et al. (1988)	Longitudinal, observational	U.S.	To perform a longitudinal study in highly motivated lactating women, focusing particularly on the first 14 days postpartum to gain a better understanding of the relationship between milk transfer during the initiation of lactation and later lactational performance.	Thirteen term infants who were breastfed, with solids introduced between 4 and 9 months of age and with formula used occasionally after 4 months in three mother–infant pairs.	EBF frequency
Nielsen et al. (2011)	Longitudinal, observational	Scotland	To test whether and how human lactation and breastfeeding practices can adapt to fulfil infant energy requirements during exclusive breastfeeding for 6 months.	Of the 47 infants, 36 infants provided data at 15.4 ± 1.3 weeks, and 38 infants at 24.5 ± 1.3 weeks. At the second time point, six infants were reported to have received complementary foods, however, there were no statistically significant differences in milk intake between these infants and those who were exclusively breastfed.	EBF volume, EBF frequency
Nommsen et al. (1991)	Longitudinal, observational	U.S.	To examine factors, including maternal anthropometric indicators, dietary intake data from a subgroup of the participants, and mother–infant variables such as nursing frequency, feed duration and milk volume, that are potentially related to interindividual differences in the amount of protein, lactose, lipid and energy in milk.	Ninety‐two mother–infant pairs were initially recruited, of which 73, 60, 50 and 46 term infants provided data at 3, 6, 9 and 12 months postpartum, respectively. Mothers of the infant had planned not to introduce solid foods before 4 months of age, or to feed >120 ml/day of other milk or formula throughout the first 12 months. The mother–infant pairs were part of the larger Davis Area Research on Lactation, Infant Nutrition and Growth (DARLING) study, designed to document total nutrient intakes and growth patterns of breastfed and formula‐fed infants during the first 18 months of life.	EBF frequency, PBF frequency
Novotny and Mata (1983)	Cross‐sectional, observational	Costa Rica	To examine the breast milk consumption and anthropometric status of rural Costa Rican infants.	Twenty term breastfeeding infants, of which ten infants were fully breastfed and the remaining ten infants were partially breastfed. Data from the infants were collected at a range of ages, including 2 to 103 days and 26 to 184 days, for the fully breastfed and partially breastfed infants, respectively.	EBF volume, EBF frequency
O'Connor et al. (2008)	Longitudinal, randomized controlled	Canada	To determine whether mixing a multinutrient fortifier to approximately one half of the human milk fed each day for a finite period after discharge improves the nutrient intake and growth of predominately human milk‐fed LBW infants through a pilot study.	Thirty‐nine preterm infants (born <33 weeks GA, 750–1,800 g; control group: 47% male, intervention group: 74% male) randomly assigned to the control group where infants were discharged from the NICU on unfortified human milk (*n* = 20), or to the intervention group receiving half their volume of human milk as nutrient‐enriched feedings after hospital discharge (*n* = 19). Infants were followed on study day 1, and 4, 8 and 12 weeks after discharge; and 6 and 12 months CA in a follow‐up study by Aimone et al. (2009).	PBF volume
Oras et al. (2015)	Longitudinal, observational	Sweden	To describe breastfeeding patterns in preterm infants up to 1 year of CA.	Eighty‐three exclusively breastfed preterm infants (born 28–33 weeks GA, 740–2,920 g; 63% male) who along with their mothers, were part of a larger study on kangaroo mother care, where a breastfeeding diary was sent home after discharge from hospital, and at 2, 6 and 12 months of the infant's CA.	EBF frequency, PBF frequency
Pao et al. (1980)	Longitudinal, observational	U.S.	To describe milk consumption and total dietary intake of completely and partially breast‐fed infants and identify factors related to these patterns.	Of the 22 term infants studied at 1, 3, 6 and 9 months, seven infants were completely breastfed at 1 month, one infant was completely breastfed at 3 months, and three infants were partially breastfed at 9 months.	EBF frequency, PBF frequency
Paul et al. (1988)	Longitudinal, observational	U.K.	To measure the growth, energy and nutrient intake longitudinally throughout infancy and to investigate the factors influencing breast milk intake. Additionally, to explore the relationship between breast milk intake and growth and provide fuller details of breast milk intake and anthropometry from 2 to 10 months of age.	Forty‐eight term infants (58% male) who received breast milk up until at least 4 months of age. At approximately 7, 8 and 10 months of age, all infants were no longer exclusively breastfed and were partially feeding.	EBF frequency, PBF frequency
Quandt (1986)	Longitudinal, observational	U.S.	To determine the range of variation in individual breastfeeding behaviours known to have biological and cultural significance, and whether these behaviours are patterned; if patterns exist, whether individual mothers maintain a similar pattern through the early lactation period; and whether patterns of breastfeeding behaviours are associated with outcomes such as duration of exclusive breastfeeding and time of weaning.	Sixty‐two term infants who showed evidence of well‐established exclusive breastfeeding were selected, who were followed at 4 and 8 weeks of age.	EBF frequency
Salmenpera, Perheentupa, and Siimes (1985)^a^	Longitudinal, observational	Finland	To evaluate the growth of infants exclusively breastfed and compare the growth with infants who weaned early and/or were given complementary foods.	One‐hundred‐and‐ninety‐eight term infants (born 37–42 weeks GA; 53% male) were followed up to 12 months of age.	EBF volume
Sievers, Oldigs, Dorner, and Schaub (1992); Sievers, Oldigs, Santer, and Schaub (2002)	Longitudinal, observational	Germany	To examine whether breastfed infants are able to adapt to zinc intakes that are lower than the recommended dietary allowance; what differences may exist between zinc intake, excretion and retention in breastfed infants and formula‐fed infants, and to compare zinc balances from term breastfed infants with those of term and preterm infants who were formula‐fed.	Of the breastfed and formula‐fed infants enrolled in the study, 7, 10, 9, 9 and 10 infants were of term birth and breastfed at 17, 35, 57 85 and 113 days, respectively.	EBF volume
Stuff and Nichols (1989)^a^	Longitudinal, observational	U.S.	To determine whether the ad libitum addition of solid foods to the diet of exclusively human milk‐fed infants will increase energy intake and reverse the decline in weight‐for‐age percentiles observed during the exclusive breastfeeding period.	Forty‐five healthy term infants who were exclusively breastfed for as least 16 weeks, where three groups emerged during the transition to monthly mixed feedings of human milk and solid foods. The groups were categorized according to introduction of mixed feeding at Group 1, 20 weeks; Group 2, 24 weeks; and Group 3, 28 weeks.	EBF volume, PBF volume
van Steenbergen et al. (1991)	Cross‐sectional, observational	Indonesia	To describe the feeding practices, breast milk intake and the consumption of additional food during infancy.	Seventy‐seven PBF infants providing cross‐sectional data at 37–56 weeks were studied from three villages along the island of Madura in East Java, Indonesia. Infants were given supplementary foods, such as mashed banana, and solid foods such as egg and fish, as the infants aged.	PBF frequency
van Steenbergen, Kusin, and van Rens (1981)^a^	Cross‐sectional, observational	Kenya	To report on breastfeeding behaviour, breast milk yield and breast milk composition from mothers living in a rural highland area.	Eighty‐five infants with mothers at different stages of lactation who were examined according to season. The participants of this study were part of two cross‐sectional studies on food intake of infants and toddlers by the Joint Project Machakos. Most infants began to be supplementally fed with some cow's milk at 3 months of age.	EBF volume, PBF volume
Yamauchi and Yamanouchi (1990)	Longitudinal, observational	Japan	To investigate the factors contributing to the frequency of breastfeeding during the first 24 hours after birth and the neonatal response to breastfeeding frequency.	Two‐hundred‐and‐ten healthy term (born 37–44 weeks GA, 2,525–4,030 g; 44% male) breastfed newborns who were observed during the first 24 hr after birth at their hospital stay.	EBF frequency

Abbreviations: CA: corrected age; EBF: exclusively breastfeeding; ELBW: extremely low birth weight; GA: gestational age; IDDM: insulin‐dependent diabetes mellitus; LBW: low birthweight; NICU: neonatal intensive care unit; PBF: partially breastfeeding.

a Data not originally available from the studies were supplemented with data presented in the review by Arcus‐Arth et al., 2005.

### 3.1 Volume of human milk intake

**Figure 2:**
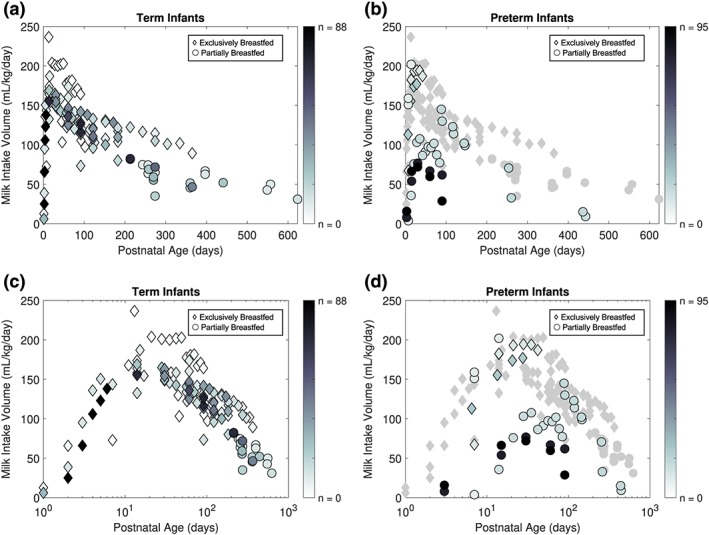
Daily weight‐normalized human milk intake (WHMI) of term and preterm infants by age. Each data point represents the mean WHMI of the study's reported age group. Intensity of marker shade corresponds to the sample size of the mean data point. (a) WHMI of exclusively breastfeeding (of all ages) and partially breastfeeding term infants (>6 months of age). (b) Exclusively breastfeeding and partially breastfeeding preterm infants with mean WHMI across postnatal ages superimposed on term infant data. (c) WHMI of term infants across log transformed postnatal age. (d) Preterm WHMI over log transformed postnatal age superimposed on term infant data

Twenty‐eight and seven studies reported on the WHMI for term and preterm infants, respectively. The mean WHMI of term infants EBF (of all ages) and PBF (>6 months of age) according to age in days are presented in Appendix B. Table 2 reports the WHMI of preterm infants exclusively and partially breastfed across all PNAs. Figure 2 shows mean WHMI plotted against PNA and log‐transformed PNA for term (Figures 2a and 2c) and preterm (Figures 2b and 2d) infants.

**Table 2:** Daily weight‐normalized human milk intakes for preterm infants

Study	Setting and population	Feeding protocol	Volume of intake^a^
Aimone et al. (2009)	Setting: Postdischarge GA at birth (weeks): Control group: 29.8 ± 1.7 Intervention group: 28.9 ± 1.2 Birth weight (g): Control group: 1322 ± 332 Intervention group: 1253 ± 242	One day prior to discharge, infants were randomized to either an intervention or control group. The control group was discharged home on unfortified human milk whereas the intervention group received nutrient enrichment of human milk. A detailed feeding protocol can be found in the description for O'Connor et al. (2008).	Control group, receiving unfortified milk:
			At 6 months CA (*n* = 17) Enteral human milk intake (ml/kg/day): 70.6 ± 43.6 Proportion of all milk feeds (%): 69 ± 38
			At 12 months CA (*n* = 16) Enteral human milk intake (ml/kg/day): 15.1 ± 23.9 Proportion of all milk feeds (%): 31 ± 46
			Intervention group, receiving fortified milk:
			At 6 months CA (*n* = 17) Enteral human milk intake (ml/kg/day): 32.8 ± 15.6 Proportion of all milk feeds (%): 43 ± 46
			At 12 months CA (*n* = 14) Enteral human milk intake (ml/kg/day): 9.1 ± 21.4 Proportion of all milk feeds (%): 22 ± 39
Atkinson et al. (1981)	Setting: NICU GA at birth (weeks): 28.3 Birth weight (g): 970	Available feeding regimens were pooled breast milk, mother's own milk and formula. Infants received a daily multivitamin. Infants started on oral feedings within the first 48 hr of life, having received only dextrose and electrolytes by IV prior to entry into the study. Milk intakes were increased as tolerated to a maximum of 180–200 ml/kg/day and fed by intermittent nasogastric gavage every 2 hr. Infants received formula if mothers' own milk supply diminished.	Pooled breast milk group:
			At 1 week PNA (*n* = 8) Human milk intake (ml/kg/day): 151 ± 15 Total fluid intake (ml/kg/day): 210 ± 10
			At 2 weeks PNA (*n* = 8) Human milk intake (ml/kg/day): 202 ± 9 Total fluid intake (ml/kg/day): 211 ± 8
			Preterm mother's milk group: At 1 week PNA (*n* = 8) Human milk intake (ml/kg/day): 159 ± 10 Total fluid intake (ml/kg/day): 184 ± 11 At 2 weeks PNA (*n* = 8) Human milk intake (ml/kg/day): 182 ± 6 Total fluid intake (ml/kg/day): 182 ± 6
			Total fluid intake include fluid of milk, IV dextrose, water and formula if the mothers' milk supply was diminished.
Cabrera Lafuente et al. (2018)	Setting: NICU GA at birth (weeks): ≤28 weeks GA group: 26.5 ± 1.4 28 to 32 weeks GA group: 30 ± 1 Birth weight (g): ≤28 weeks GA group: 905 ± 235 28 to 32 weeks GA group: 1331 ± 292	Minimal enteral feeding (20 ml/kg) with mother's own milk, or preterm infant formula if mother's own milk was not available, begun if the infant was stable on day 2 of life. Advancement pace of enteral feeding was 10–20 ml/kg/day, as tolerated, according to the unit's feeding protocol. Standardized human milk fortifiers were added when oral feeding reached 100 ml/kg/day. PN was stopped when enteral feeding reached 120 ml/kg/day.	≤28 weeks GA group (*n* = 81)
			At 3 days PNA Mother's own milk intake (ml/kg/day): 7.9 ± 0.1 Proportion of enteral intake (%): 68.7 ± 2.3
			At 15 days PNA Mother's own milk intake (ml/kg/day): 53.9 ± 5.6 Proportion of enteral intake (%): 82.2 ± 2.1
			At 30 days PNA Mother's own milk intake (ml/kg/day): 76.8 ± 6.9 Proportion of enteral intake (%): 76.2 ± 3.8
			At 60 days PNA Mother's own milk intake (ml/kg/day): 66.8 ± NR Proportion of enteral intake (%): 62 ± 3.6 At 90 days PNA Mother's own milk intake (ml/kg/day): 61.8 ± 8.6 Proportion of enteral intake (%): 51.4 ± 4.5
			28 to 32 weeks GA group (*n* = 95)
			At 3 days PNA Mother's own milk intake (ml/kg/day): 15.8 ± 1.7 Proportion of enteral intake (%): 47.1 ± 3.1
			At 3 days PNA Mother's own milk intake (ml/kg/day): 15.8 ± 1.7 Proportion of enteral intake (%): 47.1 ± 3.1
			At 15 days PNA Mother's own milk intake (ml/kg/day): 66.3 ± 5.0 Proportion of enteral intake (%): 68.0 ± 3.3
			At 30 days PNA Mother's own milk intake (ml/kg/day): 71.9 ± 5.2 Proportion of enteral intake (%): 59.0 ± 3.8
			At 60 days PNA Mother's own milk intake (ml/kg/day): 59.7 ± 8.8 Proportion of enteral intake (%): 50.9 ± 3.9
			At 90 days PNA Mother's own milk intake (ml/kg/day): 28.7 ± 11.5 Proportion of enteral intake (%): 35.8 ± 6.1

Carnielli et al. (1996)	Setting: NICU GA at birth (weeks): 29.8 ± 2.4 Birth weight (g): 1180 ± 290	IV fluids (5% dextrose in water) were started if the gastrointestinal tolerance of infant did not allow a sufficient fluid intake or if blood glucose <2.5 mmol/L. Infants did not receive PN.	At 6‐7 days PNA (*n* = 22) Milk intake (ml/kg/day): 113.0 ± 27.6
			At 13‐14 days PNA (*n* = 22) Milk intake (ml/kg/day): 155.4 ± 17.7
			At 20‐21 days PNA (*n* = 22) Milk intake (ml/kg/day): 173.8 ± 9.2
			At 27‐28 days PNA (*n* = 22) Milk intake (ml/kg/day): 177.0 ± 9.7
			Milk intake included mother's own milk and/or donor milk when mother's milk was insufficient.
Hendrickse et al. (1984)	Setting: NICU GA age at birth (weeks): 30 Birth weight (g): NR, however, infants were LBW and <1500 g at birth.	Infants fed nasogastrically or nasojejunally as much milk as they would tolerate. Maximum volume offered on the first day was 90 ml/kg and increased by 30 ml/kg/day in a stepwise fashion. Infants in the own mother's expressed breast milk group fed predominantly breast milk. These infants received additional breast milk from donor milk, where necessary. In the event that donor milk was unavailable, formula was provided. Infants ceased to receive IV dextrose after the third week.	Own mother's expressed breast milk group (*n* = 10):
			At 1 week PNA Total volume taken orally (ml/kg/day): 67.3
			At 2 weeks PNA Total volume taken orally (ml/kg/day): 182.5
			At 3 weeks PNA Total volume taken orally (ml/kg/day): 193.1
			At 4 weeks PNA Total volume taken orally (ml/kg/day): 194.4
			At 5 weeks PNA Total volume taken orally (ml/kg/day): 194.0
			At 6 weeks PNA Total volume taken orally (ml/kg/day): 187.0
Itabashi et al. (1999)	Setting: NICU GA at birth (weeks): 26.7 ± 1.4 Birth weight (g): 879.6 ± 108.2	IV fluids with glucose and electrolytes started immediately at birth and continued until day 3. On day 4, peripheral PN intake with amino acids and lipids are started and PN intake was gradually increased. Mother's own milk was fed enterally as soon as possible if the infant was stable. Formula was used if mother's milk could not be given. Phosphorus was added to the mother's own milk until fortified human milk was started. PN was discontinued when the infant tolerated 100‐120 ml/kg/day. Human milk fortifier supplemented mother's milk if amount of preterm milk was >50% of enteral feeding. Vitamin D metabolites supplemented milk when the milk intake >50 ml/kg/day.	At 1 week PNA (*n* = 6) PN (ml/kg/day): 84.4 ± 13.2 Enteral human milk (ml/kg/day): 3.7 ± 3.6 Total enteral intake (ml/kg/day): 4.2 ± 3.6
			At 2 weeks PNA (*n* = 13) PN (ml/kg/day): 84 ± 25.7 Enteral human milk (ml/kg/day): 35.7 ± 22.7
			Total enteral intake (ml/kg/day): 39.1 ± 23.1 At 3 weeks PNA (*n* = 15) PN (ml/kg/day): 34.4 ± 28.1 Enteral human milk (ml/kg/day): 75.9 ± 36.1
			Total enteral intake (ml/kg/day): 87.7 ± 34.1 At 4 weeks PNA (*n* = 15) PN (ml/kg/day): 9.4 ± 12.2 Enteral human milk (ml/kg/day): 103.1 ± 39.9
			Total enteral intake (ml/kg/day): 117.4 ± 26.5 At 5 weeks PNA (*n* = 15) PN (ml/kg/day): 11.1 ± 16.9 Enteral human milk (ml/kg/day): 107.6 ± 42.3
			Total enteral intake (ml/kg/day): 127.8 ± 27 At 6 weeks PNA (*n* = 15) PN (ml/kg/day): 7.7 ± 14 Enteral human milk (ml/kg/day): 86.4 ± 59.9
			Total enteral intake (ml/kg/day): 134.5 ± 21.5 At 7 weeks PNA (*n* = 12) PN (ml/kg/day): 0.6 ± 1.4 Enteral human milk (ml/kg/day): 91.2 ± 65.1
			Total enteral intake (ml/kg/day): 145.7 ± 6.9 At 8 weeks PNA (*n* = 12) PN (ml/kg/day): 3.1 ± 6.7 Enteral human milk (ml/kg/day): 97.4 ± 60.4
			Total enteral intake (ml/kg/day): 139.8 ± 14.7 At 9 weeks PNA (*n* = 14)
			PN (ml/kg/day): 3.2 ± 8.9 Enteral human milk (ml/kg/day): 96.4 ± 65.4 Total enteral intake (ml/kg/day): 140.9 ± 21.1 At 10 weeks PNA (*n* = 14)

			PN (ml/kg/day): 0 Enteral human milk (ml/kg/day): 101.8 ± 60.2 Total enteral intake (ml/kg/day): 145.5 ± 9.6
			At 11 weeks PNA (*n* = 14) PN (ml/kg/day): 0 Enteral human milk (ml/kg/day): 87.4 ± 63.8 Total enteral intake (ml/kg/day): 146.8 ± 11.2
			At 12 weeks PNA (*n* = 14) PN (ml/kg/day): 0 Enteral human milk (ml/kg/day): 77.3 ± 65.8 Total enteral intake (ml/kg/day): 148.9 ± 9.8

O'Connor et al. (2008)	Setting: Post‐discharge GA at birth (weeks): Control group: 29.8 ± 1.7 Intervention group: 28.9 ± 1.2 Birth weight (g): Control group: 1322 ± 332 Intervention group: 1253 ± 242	Daily iron supplement and vitamin drops after discharge; however, those in the intervention group did not receive vitamins A and C. One day prior to discharge, infants who were randomly assigned to the control group were discharged from the hospital on unfortified human milk. Infants randomly assigned to the intervention group had half the volume of human milk enriched with a powdered multinutrient human milk fortifier. Remaining feedings were provided as unfortified milk. Families chose when during the day they wished to provide the nutrient‐enriched feedings and use of a bottle or supplemental nursing system. Infants in the control group who demonstrated poor intake and growth received nutrient enrichment under the discretion of the infants' pediatrician (e.g., powdered postdischarge formula added to human milk).	Control group, receiving unfortified milk:
			At 4 weeks post‐discharge (*n* = 16) Total human milk (ml/kg/day): 145 ± 46 Total intake (ml/kg/day): 155.9 ± 28.3
			At 8 weeks post‐discharge (*n* = 17) Total human milk (ml/kg/day): 123 ± 45 Total intake (ml/kg/day): 146.2 ± 24.3
			At 12 weeks post‐discharge (*n* = 17) Total human milk (ml/kg/day): 102 ± 46 Total intake (ml/kg/day): 134.1 ± 32.7
			Intervention group, receiving fortified milk:
			At 4 weeks post‐discharge (*n* = 17) Total human milk (ml/kg/day): 130 ± 45 Total intake (ml/kg/day): 146.7 ± 34.3
			At 8 weeks post‐discharge (*n* = 15) Total human milk (ml/kg/day): 114 ± 26 Total intake (ml/kg/day): 121.7 ± 25.8
			At 12 weeks post‐discharge (*n* = 15) Total human milk (ml/kg/day): 99 ± 24 Total intake (ml/kg/day): 110.6 ± 20.3
			Total human milk includes milk at the breast, and unfortified and fortified milk. Total intake includes nutrients from all sources, including human milk and formula.

Abbreviations: CA: corrected age; GA: gestational age; IV: intravenous; NICU: neonatal intensive care unit; NR: not reported; PN: parenteral nutrition; PNA: postnatal age; *SD*: standard deviation.

a Presented as mean ± *SD*, or only as mean if *SD* was not available.

**Figure 3:**
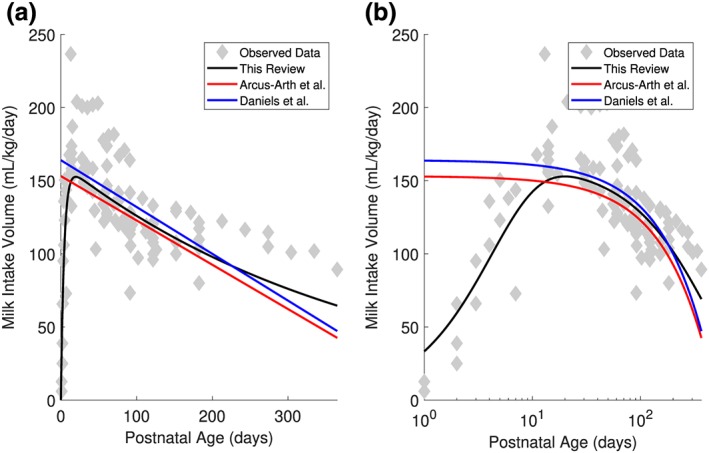
Regression equations describing the mean weight‐normalized human milk intake (WHMI) of exclusively breastfeeding term infants across postnatal age, with postnatal age untransformed (a) and log‐transformed (b). Data are presented from this review, Arcus‐Arth et al. (2005) and Daniels et al. (2019)

Across all studies, mean WHMI increases from birth until reaching a maximum of 152.6 ml/kg/day at 19.7 days of age, and then declines thereafter (Figure 2a). Figure 3 presents the results of nonlinear regression modelling on the WHMI for EBF term infants over PNA (Figure 3a) and log‐transformed PNA (Figure 3b). The modelling resulted in the following optimized parameter values, with no evidence of correlations between the parameters: *θ*_1_ = 160.39, *θ*_2_ = 0.232 and *θ*_3_ = 0.00252. The equation describing the mean WHMI of EBF term infants is as follows:

$$\text{WHMI}\;\left(\text{ml/kg/day}\right)=160.39\cdot \frac{0.232}{0.232-0.00252}\cdot \left(e^{-0.00252\cdot t}-e^{-0.232\cdot t}\right).$$

The linear regression equations using individual subject data from five studies acquired by Arcus‐Arth et al. (2005) and a single study by Daniels et al. (2019) are shown in Figures 3a and 3b for comparison.

In comparing the WHMI between preterm and term infants, Figures 2b and 2d show similar WHMI among EBF preterm and EBF term infants from 7 to 28 days PNA; PBF preterm and EBF term infants from 7 to 14 days PNA, and 88 to 146 days PNA; and PBF preterm and PBF term infants from 254 to 443 days PNA. Of the studies reporting on the milk intake of preterm infants described in Table 2, two provided WHMI volumes from EBF preterm infants in the neonatal intensive care unit (NICU) setting (Carnielli et al., 1996; Hendrickse, Spencer, Roberton, & Hull, 1984). The remaining studies with milk intake at the NICU presented preterm infants PBF with mother's own milk or donor human milk and preterm formula (Atkinson, Bryan, & Anderson, 1981; Cabrera Lafuente et al., 2018; Itabashi, Miura, Okuyama, Takeuchi, & Kitazawa, 1999). Two studies included infants who were also parenterally fed, which is reflected in their substantially lower WHMI as compared with those of term infants (Cabrera Lafuente et al., 2018; Itabashi et al., 1999; Figure 2d). No studies informed EBF preterm infants after NICU discharge. O'Connor et al. (2008) and Aimone et al. (2009) reported intake volume data on PBF preterm infants after discharge, where the latter followed infants from the randomized controlled trial by O'Connor et al. (2008) up to 12 months CA.

### 3.2 Frequency of human milk feeding

**Figure 4:**
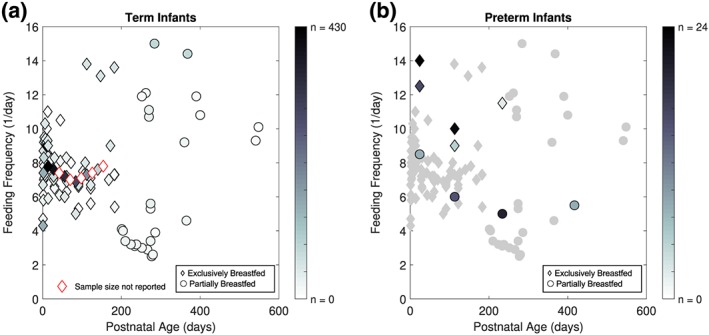
Daily frequency of human milk feeding of term and preterm infants by age. (a) Human milk feeding frequency of term infants by the mean number of feeds per day of each study's reported age group. (b) Exclusively and partially breastfed preterm infants with human milk feeding frequency across postnatal ages superimposed on term infant data. Data are presented from a single study by Oras et al. (2015) and as median number of feeds per day

Twenty‐four studies and one study reported on the frequency of human milk intake throughout the day for term and preterm infants, respectively. The mean feeding frequencies of term and preterm infants EBF and preterm infants PBF according to PNA in days are presented in Appendix C. There were no studies reporting on the feeding frequencies of term infants past 6 months of age. Figure 4a presents the daily feeding frequencies of term infants plotted against PNA. Feeding frequency in term infants increased until approximately 10 days of age and was relatively stable thereafter. The average feeding frequency across all ages of EBF term infants, weighted by sample size in each dataset, was 7.7 feeds/day (range: 4.3 to 13.8 feeds/day). The mean feeding frequencies at 16, 21, and 26 weeks PNA from a single study (Cohen, Brown, Canahuati, Rivera, & Dewey, 1994) were much greater than values of other EBF term infants. PBF term infants tended to either feed at a high (>9 feedings/day) or low frequency (<6 feedings/day). Figure 4b shows the feeding frequency of preterm infants from a single study by Oras et al. (2015). The study reported the frequency of EBF preterm infants postdischarge from the NICU directly feeding at the breast, or at the breast and expressed milk (from the tube, cup and/or bottle feeding), were 12.5 and 14 feeds/day, respectively. At 2 months CA, the infants were feeding at the breast 9 feeds/day and at the breast with expressed milk 10 feeds/day. At 6 months CA, infants who were consuming expressed milk were fed 11.5 feeds/day.

As shown in Figure 4, EBF preterm infants tend to feed at a greater frequency than preterm infants who were PBF (Figure 4a) and the average feeding frequency of term infants (Figure 4b). Conversely, PBF preterm infants were feeding at a lower frequency than the average for term infants, except at post‐discharge (Figure 4b).

## 4 DISCUSSION

In this review, human milk feeding parameters of daily WHMI and frequency of feeds as a function of age were quantified for term and preterm infants with literature data for inputs into PBPK models designed for drug‐in‐milk risk assessment. This review is the first to perform a literature search to report on daily WHMI and feeding frequencies for preterm infants, and feeding frequencies for term infants. Through the literature search process, weight‐adjusted intake values were obtained and reported for term and preterm infants. For term infants, a regression model described average volume intake levels in those exclusively breastfed from birth to 1 year of age. Frequency of feeding was described for both term and preterm infants across PNAs.

The rate of human milk consumption by term infants from approximately 20 days to 6 months PNA was consistent with published analyses that also examined WHMI (Figure 3). Linear regression models by Arcus‐Arth et al. (2005) and Daniels et al. (2019) appeared to predict similar rates of WHMI as the nonlinear regression model of this review until 6 months of age. Data past 6 months of age were not comparable due to the inclusion of only EBF infants >6 months of age in this review, whereas Arcus‐Arth et al. (2005) included PBF infants >6 months of age and Daniels et al. (2019) modelled intake from a cross‐sectional study of 113 infants with data only until 5–6 months of age. As a strength of this review, WHMI of EBF term infants were captured by the regression model for >6 months of age and will therefore provide conservative values for subsequent risk assessments. A maximum mean intake of 152.6 ml/kg/day at 19.7 days of age was identified. This suggests that EBF term infants are at greatest risk of drug exposure at 2–4 weeks of age. These results are consistent with a review by Anderson and Valdes (2015), reporting a maximum average volume of intake between 170 and 184 ml/kg/day at 4 to 35 days of age from a longitudinal study in 13 lactating women (Neville et al., 1988). In a review by McNamara and Abbassi (2004), the reported peak intake volume was approximately 173.8 ml/kg/day at 1 month of age from four studies (Butte, Garza, Stuff, Smith, & Nichols, 1984; Hopkinson, Schanler, Fraley, & Garza, 1992; Neville, 1999; Rattigan, Ghisalberti, & Hartmann, 1981). Furthermore, the increased risk at 2–4 weeks of age is reflected in a review of case reports and studies of adverse reactions in breastfed infants of mothers taking medication. In their review, approximately two thirds of reported adverse reactions occurred during the first month PNA, and more than three quarters occurred in the first 2 months PNA (Anderson, Manoguerra, & Valdes, 2016).

WHMI of EBF preterm infants (7–28 days PNA) and PBF preterm infants (7–14 days, 88–146 days PNA and 254–443 days PNA), who did not receive parenteral nutrition, were comparable to that of term infants (Figures 2b and 2d). The proportion of human milk consumption from all enteral intake appeared to dictate whether EBF and PBF preterm infants, who did not receive parenteral nutrition, approximated the WHMI of EBF or PBF term infants. However, more data regarding the proportion of breast milk intake by PBF term infants would be necessary to confirm these findings. Nevertheless, the results suggest that preterm infants do not present a substantial difference in weight‐normalized feeding volume trajectory across ages as compared with term infants, as one might expect from their late development of suck‐swallow‐breath coordination and mother's delayed onset of lactogenesis. Controlled enteral feeds (e.g., NG tubes) dictated by hospital protocols to reach target volumes and introduction of donor human milk when mother's own milk is unavailable may contribute to the strong observed WHMI. The observation that preterm infants are able to feed at similar WHMI as term infants has important implications. With similar weight‐normalized doses and lower clearance in preterm infants relative to term infants, this group is at risk for higher exposure and toxic effects of the drug.

Human milk feeding frequency of EBF term infants slightly increased in the first 10 days of life and subsequently declined and stabilized (Figure 3). Daily frequency of feeds as a function of age shown in Figure 3 suggests that feeding frequency was fairly constant over 6 months of age. In contrast, frequencies were either high (>9 feeds/day) or low (<6 feeds/day) across ages past 6 months for PBF term infants. This stark contrast between feeding frequencies could be due to differences in PBF patterns of developed and developing countries, and rural and urban communities. Studies reporting higher frequencies were conducted in developing countries or rural communities (Amatayakul et al., 1999; Martinez & Chavez, 1971; van Steenbergen, Kusin, Kardjati, & Renqvist, 1991), whereas the lower frequencies were reported in studies conducted in developed countries and urban communities (Howie, McNeilly, Houston, Cook, & Boyle, 1981; Michaelsen, Larsen, Thomsen, & Samuelson, 1994; Nommsen, Lovelady, Heinig, Lönnerdal, & Dewey, 1991; Pao, Himes, & Roche, 1980; Paul, Black, Evans, Cole, & Whitehead, 1988). Similarly, the relatively high mean feeding frequencies of 13.1 to 13.8 feeds/day in those EBF were from term infants of a rural community (Cohen et al., 1994). More research may uncover the influence of cultural practices on feeding frequency, such as time spent at home, support from family members and willingness to breastfeed.

A single study by Oras et al. (2015) reported on the frequency of human milk feeding in the preterm population. EBF preterm infants tended to feed at a greater frequency than PBF preterm infants and the average feeding frequency of term infants (Figure 4b). Conversely, PBF preterm infants tended to feed less frequently than the term infant average (Figure 4b).

This review is not the first to identify quantified milk feeding parameters as inputs into PBPK models for predicting infant drug exposures through breast milk. In fact, existing literature shows that published PBPK models have used different values of milk intake feeding parameters as inputs. Schreiber (1993) used PBPK modelling to predict infant exposure to perchloroethylene, where an infant weighing 7.2 kg was assumed to ingest 700 ml/day of breast milk for 12 months postpartum. Equivalent to an average WHMI of 97.2 ml/kg/day, infant daily dose would be largely underpredicted at 2–4 weeks to 6 months PNA according to the WHMI findings in this review (Figure 3). In one case report, obstructive jaundice and hepatomegaly were observed in an infant receiving 1.4 mg/kg/day of tetrachloroethylene (Bagnell & Ellenberger, 1977). Updating the calculations for the infant dose through breast milk with the WHMI from this review yields a daily intake of 1.3 mg/kg/day, which was previously calculated as 0.82 mg/kg/day (Schreiber, 1993). This demonstrates the potential influence of different intake values and the importance of identifying an appropriate milk volume in such risk assessments. In another study by Delaney et al. (2018), the authors used values reported by Kent et al. (2006) to simulate out the variability in feeding parameters, employing a mean ± *SD* of 76 ± 12.6 ml/feed and 11 ± 3 feeds/day. Interestingly, the calculated mean of these parameters across all age groups evaluates to 153 ml/kg/day. While generous, this metric overestimates doses for children >2 months of age. For this reason, milk intake feeding parameters that capture changes across PNA, such as the regression equation as derived from the volume of intake literature values in this review, would be an improvement.

Although mean WHMI and frequency of feeds were obtained through the literature, variation around these values was not explored in this review. Future research focusing on the variability of milk feeding parameters as inputs into the PBPK models will be important to subsequent drug‐in‐milk risk assessments. Particularly, these efforts can help identify infants who are outliers and may be at highest risk for receiving toxic effects of the drug.

## 5 CONCLUSION

In summary, the volume and frequency of human milk intake in term and preterm infants were quantified to provide dose information for paediatric PBPK models that will be used to inform infant exposure and subsequent risk assessment. The derived nonlinear regression equation of WHMI can be used to describe the volume of intake for EBF term infants. Because the WHMI of preterm infants were consistent with the observed WHMI of EBF term infants, the nonlinear regression equation may be applicable to preterm infants. For daily frequency of feeds, a weighted mean of 7.7 feeds/day can be used for EBF term infants across all ages. The data from Oras et al. (2015) provided context in preterm infant milk intake feeding frequencies, however, more data are needed to inform the frequency of feeds in this population.

## ACKNOWLEDGMENT

This study was funded by the Canadian Institutes of Health Research, grant PJT‐159782.

## CONFLICTS OF INTEREST

The authors declare that they have no conflicts of interest.

## CONTRIBUTIONS

CHTY and ANE conceived and designed the study. CHTY performed the search, screening, and extraction process. SF performed the screening. CHTY and PRVM performed the analysis of data. CHTY, SF, and PRVM contributed to writing the manuscript. All authors revised and approved the final and submitted version of the manuscript.

## Appendix A

Ovid MEDLINE(R) and Epub Ahead of Print, In‐Process and Other Non‐Indexed Citations, Daily and Versions(R) 1946 to July 02, 2019

1. exp Infant, Low Birth Weight/
2. exp Infant, Premature/
3. Premature Birth/
4. ((premature or preterm or pre‐term or full‐term) adj (infant* or neonate* or newborn* or birth*)).tw.
5. ((breastfeed* or breast‐feed* or breastfed or breast‐fed or feeding or breastmilk or breast‐milk or milk) adj3 (infant* or neonate* or newborn*)).tw.
6. 1 or 2 or 3 or 4 or 5
7. Breast Feeding/
8. Milk, Human/
9. Lactation/
10. 7 or 8 or 9
11. ((breastfeed* or breast‐feed* or breastfed or breast‐fed or feeding or breastmilk or breast‐milk or milk) adj3 (intake* or volume* or consum* or frequency or frequencies or pattern*)).tw.
12. 6 and 10 and 11
13. limit 12 to (english language and humans)

Embase 1974 to 2019 July 02

1. prematurity/
2. ((premature or preterm or pre‐term or full‐term) adj (infant* or neonate* or newborn* or birth*)).ti,ab.
3. ((breastfeed* or breast‐feed* or breastfed or breast‐fed or feeding or breastmilk or breast‐milk or milk) adj3 (infant* or neonate* or newborn*)).ti,ab.
4. 1 or 2 or 3
5. breast feeding/
6. breast milk/
7. lactation/
8. 5 or 6 or 7
9. ((breastfeed* or breast‐feed* or breastfed or breast‐fed or feeding or breastmilk or breast‐milk or milk) adj3 (intake* or volume* or consum* or frequency or frequencies or pattern*)).ti,ab.
10. 4 and 8 and 9
11. limit 10 to (human and english language)

## Appendix

**Table:**

Age (days)^a^	Study	No. of infants	WHMI volume (ml/kg/day)	*SD*
Exclusively breastfed				
1	Casey et al., 1986	3	12.6	15.5
	Evans et al., 2003	26	6	7.1
2	Casey et al., 1986	10	38.8	22.3
	Evans et al., 2003	88	25	20.6
	Novotny & Mata, 1983	1	65.7	NR
3	Casey et al., 1986	10	95.1	45.6
	Evans et al., 2003	88	66	33.8
4	Casey et al., 1986	11	135.9	49.5
	Evans et al., 2003	88	106	36.6
5	Casey et al., 1986	11	150.5	28.2
	Evans et al., 2003	88	123	42.2
6	Evans et al., 2003	88	138	36.6
7	English, 1985	1	72.8	NR
	Ferris et al., 1993; Neubauer et al., 1993	10	143.7	36.9
11	Novotny & Mata, 1983	1	167.7	NR
13	Novotny & Mata, 1983	1	236.6	NR
14	Bhutta et al., 2004	12	132.6	27.7
	English, 1985	1	158.3	NR
	Ferris et al., 1993; Neubauer et al., 1993	9	156.3	40.8
	Forsum & Sadurskis, 1986	22	168.9	38.8
	Janas et al., 1985	11	164	NR
	Krebs et al., 1994	71	155.3	29.1
	Novotny & Mata, 1983	2	173.7	49.9
15	van Steenbergen et al., 1981	7	187	29
17	Sievers et al., 1992; Sievers et al., 2002, b	7	154.4	NR
21	English, 1985	1	203.9	NR
28	English, 1985	1	201.0	NR
	Forsum & Sadurskis, 1986	22	165.0	28.2
30	Borschel et al., 1986	15	158	46.5
	Brown et al., 1986	36	163.1	29.1
	Butte, Garza, Smith, & Nichols, 1984, Butte, Garza, Stuff, et al., 1984	37	154.4	23.3
	Butte, Smith, & Garza, 1990	17	148.5	25.2
	Hofvander et al., 1982	25	149.5	24.3
35	English, 1985	1	200.0	NR
	Sievers et al., 1992; Sievers et al., 2002, b	10	145.6	NR
37	Butte et al., 1983	2	158.8	13.2
42	English, 1985	1	201.9	NR
	Forsum & Sadurskis, 1986	22	142.7	37.9
	Motil et al., 1997	10	158.3	17.5
45	Novotny & Mata, 1983	2	129.6	3.5
46	Butte et al., 1988	1	103.2	NR
49	English, 1985	1	202.9	NR
56	English, 1985	1	177.7	NR
	Forsum & Sadurskis, 1986	22	140.8	23.3
	Janas et al., 1985	11	134	NR
57	Sievers et al., 1992; Sievers et al., 2002, b	9	122.3	NR
61	Butte et al., 1983	4	126.9	35
	Bandara et al., 2015	8	160.2	29.1
	Bandara et al., 2015	8	173.8	21.4
	Borschel et al., 1986	15	128	27.1
	Brown et al., 1986	42	146.6	31.1
	Butte, Garza, Smith et al., 1984, Butte, Garza, Stuff et al., 1984	40	125.2	18.4
	Hofvander et al., 1982	25	143.7	22.3
	Michaelsen et al., 1994	60	135.9	23.3
63	English, 1985	1	180.6	NR
70	English, 1985	1	181.6	NR
	Forsum & Sadurskis, 1986	22	134.0	21.4
72	Novotny & Mata, 1983	1	122.8	NR
76	Bandara et al., 2015	8	135.9	45.6
	Bandara et al., 2015	8	141.7	16.5
	van Steenbergen et al., 1981	13	120	41
77	English, 1985	1	167.0	NR
81	Butte et al., 1988	1	121.7	NR
84	English, 1985	1	170.9	NR
	Motil et al., 1997	10	118.4	14.6
85	Sievers et al., 1992; Sievers et al., 2002, b	9	125	NR
91	English, 1985	1	164.1	NR
	Bhutta et al., 2004	12	73.3	10.2
	Brown et al., 1986	33	141.7	24.3
	Butte, Garza, Smith et al., 1984, Butte, Garza, Stuff et al., 1984	37	113.6	19.4
	Dewey et al., 1991a, 1991b	73	126.2	17.5
	Hofvander et al., 1982	25	128.2	17.5
	Krebs et al., 1994	71	116.5	15.5
92	Butte et al., 1988	1	105.6	NR
94	Butte et al., 1983	11	120	24
102	Novotny & Mata, 1983	2	97.3	22.4
108	Nielsen et al., 2011	36	137.9	16.5
112	Ettyang et al., 2005	10	115	15.8
113	Sievers et al., 1992; Sievers et al., 2002, b	10	119.4	NR
116	Butte et al., 1983	7	115.6	19.8
122	Borschel et al., 1986	15	96	27.1
	Brown et al., 1986	15	133	18.4
	Butte, Garza, Smith et al., 1984, Butte, Garza, Stuff et al., 1984	41	107.8	16.5
	Butte, Smith, & Garza, 1990	15	110.7	19.4
	Michaelsen et al., 1994	36	120.4	16.5
	Salmenpera et al., 1985	12	121.4	20.4
	Stuff & Nichols, 1989	45	110.7	17.5
	Butte et al., 1988	2	99.9	17.3
126	Motil et al., 1997	10	102.9	26.2
152	Bandara et al., 2015	9	131.1	16.5
	Bandara et al., 2015	7	107.8	28.2
	Brown et al., 1986	14	129.1	19.4
	Stuff & Nichols, 1989	26	100.0	21.4
172	Nielsen et al., 2011	38	128.2	14.6
183	Bhutta et al., 2004	12	117.3	49.2
	Borschel et al., 1986	15	80	23.2
	Brown et al., 1986	13	121.4	23.3
	Salmenpera et al., 1985	31	109.7	16.5
	Stuff & Nichols, 1989	8	103.9	26.2
213	Brown et al., 1986	12	122.3	18.4
243	Brown et al., 1986	10	116.5	17.5
274	Brown et al., 1986	5	114.6	23.3
	Salmenpera et al., 1985	16	104.9	19.4
304	Salmenpera et al., 1985	10	101.0	17.5
335	Salmenpera et al., 1985	5	101.9	16.5
365	Salmenpera et al., 1985	4	89.3	17.5
Partially breastfed				
213	Stuff & Nichols, 1989	8	81.6	26.2
	Krebs et al., 1994	71	82.5	16.5
243	Stuff & Nichols, 1989	7	74.8	25.2
257	Martinez & Chavez, 1971	9	63.7	10.2
	Martinez & Chavez, 1971	8	74.4	16.7
259	van Steenbergen et al., 1981	22	69	25.0
270	Amatayakul et al., 1999	26	55.3	20.4
	Amatayakul et al., 1999	26	59.2	17.5
274	Dewey et al., 1991a, 1991b	50	71.8	23.3
	Stuff & Nichols, 1989	7	65.0	18.4
	Michaelsen et al., 1994	18	35	21.4
360	Amatayakul et al., 1999	16	45.6	21.4
	Amatayakul et al., 1999	18	51.5	25.2
365	Dewey et al., 1991a, 1991b	42	46.6	26.2
397	Martinez & Chavez, 1971	9	62.7	9.6
	Martinez & Chavez, 1971	8	66.8	6.1
441	van Steenbergen et al., 1981	22	52	17.0
549	Martinez & Chavez, 1971	9	42.7	7.2
557	Martinez & Chavez, 1971	8	49.9	7.2
624	van Steenbergen et al., 1981	12	31	15.0

Abbreviations: NR: not reported; WHMI: weight‐normalized human milk intake.

a Age reported as weeks or months were converted into an approximate age in days (e.g., 2 weeks × 7 days/week = 14 days, 5 months × (365 days/12 months) = 152 days).

b Data are reported as median WHMI. All other data are presented as mean WHMI.

## Appendix

**Table:**

PNA (days)^a^	Study	No. of infants	Feeds per day	*SD*
Exclusively breastfed term infants				
1	Yamauchi & Yamanouchi, 1990	140	4.3	2.5
	de Carvalho et al., 1982	46	6.7	2.8
	Houston et al., 1983	18	4.7	1.2
2	Ferris et al., 1993; Neubauer et al., 1993	11	6.1	1.4
	Yamauchi & Yamanouchi, 1990	140	7.4	3.9
	de Carvalho et al., 1982	46	8.7	3.1
	Novotny & Mata, 1983	1	8	NR
	Houston et al., 1983	18	5.8	1.2
3	Ferris et al., 1993; Neubauer et al., 1993	11	7.8	1.9
	Jia et al., 2018	71	8.3	2.1
	Houston et al., 1983	18	7	0.9
	de Carvalho et al., 1982	46	9.2	2.7
4	Houston et al., 1983	18	7.1	0.9
	de Carvalho et al., 1982	46	9.5	3.3
5	Houston et al., 1983	18	7.3	1.4
	de Carvalho et al., 1982	46	9.3	2.7
6	de Carvalho et al., 1982	46	10.3	3.4
7	Ferris et al., 1993; Neubauer et al., 1993	11	8.2	1.4
	de Carvalho et al., 1982	46	9.4	3.0
8	de Carvalho et al., 1982	46	9.1	2.5
9	de Carvalho et al., 1982	46	9.0	3.1
10	Jia et al., 2018	45	8.5	1.2
	de Carvalho et al., 1982	46	9.2	2.9
11	Neville et al., 1988	12	7.5	3.8
	Novotny & Mata, 1983	1	10	NR
	de Carvalho et al., 1982	46	9.0	3.0
12	de Carvalho et al., 1982	46	9.0	3.1
13	Novotny & Mata, 1983	1	11	NR
	de Carvalho et al., 1982	46	9.3	3.0
14	Ferris et al., 1993; Neubauer et al., 1993	11	8.4	2.6
	Hornell et al., 1999^b^	430	7.8	NR
	Howie et al., 1981	27	5.9	1.0
	Novotny & Mata, 1983	2	8.5	0.7
	de Carvalho et al., 1982	46	9.0	2.6
22	Neville et al., 1988	12	8.2	2.8
28	Hornell et al., 1999 ^b^	395	7.6	NR
	Matheny & Picciano, 1986	37	7	1.6
	Kent et al., 2013	52	7.6	NR
	Quandt, 1986	62	7.2	1.7
30	Borschel et al., 1986	15	6.5	1.2
	Butte et al., 1990	10	7.2	1.6
	Butte, Garza, Smith et al., 1984, Butte, Garza, Stuff et al., 1984	37	8.3	1.9
	Pao et al., 1980	7	6.6	1.1
42	Hornell et al., 1999 ^b^	NR	7.4	NR
43	Paul et al., 1988	20	6.1	1.0
45	Novotny & Mata, 1983	2	10.5	3.5
	Neville et al., 1988	13	8.1	2.2
56	Hornell et al., 1999 ^b^	337	7.2	NR
	Matheny & Picciano, 1986	37	6	1.6
	Quandt, 1986	48	7.1	1.6
60	Jia et al., 2018	25	8.3	1.0
61	Borschel, Kirskey, & Hannemann, 1986	15	6.3	0.8
	Michaelsen et al., 1994	60	6.9	1.8
	Butte, Garza, Smith et al., 1984, Butte, Garza, Stuff et al., 1984	40	7.2	1.9
70	Hornell et al., 1999 ^b^	NR	7	NR
72	Novotny & Mata, 1983	1	8	NR
84	Hornell et al., 1999 ^b^	290	6.9	NR
	Matheny & Picciano, 1986	47	5	1.7
91	Kent et al., 2013	52	6.6	NR
	Nommsen et al., 1991	58	6.7	1.5
	Butte, Garza, Smith et al., 1984, Butte, Garza, Stuff et al., 1984	37	6.8	1.9
	Pao et al., 1980	1	5.3	NR
98	Hornell et al., 1999 ^b^	NR	7.1	NR
102	Novotny & Mata, 1983	2	7.5	2.1
105	Neville et al., 1988	13	7.3	1.8
108	Nielsen et al., 2011 ^b^	43	8	NR
112	Hornell et al., 1999 ^b^	189	7.3	NR
	Cohen et al., 1994	50	13.8	3
122	Borschel et al., 1986	15	5.6	1.5
	Michaelsen et al., 1994	36	7.1	1.9
	Butte et al., 1990	10	6.5	1.1
	Butte, Garza, Smith et al., 1984, Butte, Garza, Stuff et al., 1984	41	6.7	1.8
126	Hornell et al., 1999 ^b^	NR	7.4	NR
140	Hornell et al., 1999 ^b^	79	7.6	NR
147	Cohen et al., 1994	50	13.1	2.7
154	Hornell et al., 1999 ^b^	NR	7.8	NR
168	Hornell et al., 1999 ^b^	20	7	NR
172	Nielsen et al., 2011 ^b^	41	9	NR
182	Hornell et al., 1999 ^b^	7	7.3	NR
	Cohen et al., 1994	50	13.6	1.8
183	Borschel et al., 1986	15	5.4	1.2
	Pao et al., 1980	1	7.3	NR
Partially breastfed term infants				
201	Paul et al., 1988	16	4.1	2.0
204	Paul et al., 1988	21	4.0	1.7
210	Howie et al., 1981	22	3.4	1.4
224	Howie et al., 1981	21	3.2	1.3
234	Paul et al., 1988	12	3.1	1.7
237	Paul et al., 1988	18	3.2	2.1
238	Howie et al., 1981	19	3.2	1.2
252	Howie et al., 1981	17	3.0	1.3
	Martinez & Chavez, 1971	9	11.9	1.2
262	Martinez & Chavez, 1971	8	12.1	1.7
266	Howie et al., 1981	15	2.9	1.5
270	Amatayakul et al., 1999	26	10.7	3.9
	Amatayakul et al., 1999	26	11.1	2.8
274	Michaelsen et al., 1994	18	3.4	1.7
	Pao et al., 1980	3	5.3	0.9
	Nommsen et al., 1991	28	5.6	1.7
277	Paul et al., 1988	12	2.5	2.5
280	Howie et al., 1981	15	2.6	0.6
284	van Steenbergen et al., 1991	77	15.0	1.6
286	Paul et al., 1988	7	3.9	2.9
360	Amatayakul et al., 1999	16	9.2	4.8
	Amatayakul et al., 1999	18	9.2	2.8
365	Nommsen et al., 1991	21	4.6	2.1
368	van Steenbergen et al., 1991	77	14.4	2
390	Martinez & Chavez, 1971	9	11.9	1.4
400	Martinez & Chavez, 1971	8	10.8	1.9
541	Martinez & Chavez, 1971	9	9.3	0.9
548	Martinez & Chavez, 1971	8	10.1	0.8
Exclusively and partially breastfed preterm infants				
24	Oras et al., 2015 ^b^	24	14	NR
24	Oras et al., 2015 ^b^	16	12.5	NR
24	Oras et al., 2015 ^b,c^	8	8.5	NR
113	Oras et al., 2015 ^b^	23	10	NR
113	Oras et al., 2015 ^b^	5	9	NR
113	Oras et al., 2015 ^b,c^	15	6	NR
234	Oras et al., 2015 ^b^	2	11.5	NR
234	Oras et al., 2015 ^b,c^	20	5	NR
417	Oras et al., 2015 ^b,c^	8	5.5	NR

Abbreviations: NR: not reported; PNA: postnatal age.

a Age reported as weeks or months were converted into an approximate age in days (e.g., 2 weeks × 7 days/week = 14 days, 5 months × (365 days/12 months) = 152 days).

b Median number of feeds per day. All other studies present mean number of feeds per day.

c PBF data from preterm infants. All other feeding frequency data for preterm infants are presented for EBF.
